# Concurrent Graves' Disease and TSH Secreting Pituitary Adenoma Presenting Suppressed Thyrotropin Levels: A Case Report and Review of the Literature

**DOI:** 10.3389/fendo.2020.00523

**Published:** 2020-08-06

**Authors:** Jinrong Fu, Anhua Wu, Xiaoli Wang, Haixia Guan

**Affiliations:** ^1^Department of Endocrinology and Metabolism, Institute of Endocrinology, The First Hospital of China Medical University, Shenyang, China; ^2^Department of Neurosurgery, The First Hospital of China Medical University, Shenyang, China; ^3^Department of Endocrinology, Guangdong Provincial People's Hospital, Guangdong Academy of Medical Science, Guangzhou, China

**Keywords:** TSH secreting pituitary adenoma, TSHoma, Graves' disease, concomitant, thyrotropin

## Abstract

**Background:** Thyroid stimulating hormone (TSH) secreting pituitary adenoma (TSHoma) is a rare cause of hyperthyroidism. To date there have been only thirteen cases reporting the coexistence of TSHoma with Graves' disease (GD). The diagnosis and management for such hyperthyroidism due to both etiologies remain challenging.

**Case Report:** A 55-year-old Chinese female presented with signs and symptoms of thyrotoxicosis. Thyroid function tests showed elevated thyroid hormones and mildly suppressed TSH values. Her anti-thyrotropin receptor antibody (TRAb) was positive. Octreotide suppression test successfully decreased her TSH. Magnetic resonance imaging showed a pituitary macroadenoma. She underwent endoscopic trans-sphenoidal resection and surgical pathology confirmed a TSH producing pituitary adenoma. Methimazole was prescribed after surgery and her clinical course was monitored.

**Conclusions:** Here we report a case of a 55-year-old female with TSHoma and Graves' disease whose TSH level was mildly suppressed. This case emphasizes the importance of thoroughly evaluating the thyroid function test during the diagnosis of hyperthyroidism. It also highlights the challenges in the diagnosis and treatment of this rare condition.

## Introduction

Hyperthyroidism is a pathological disorder caused by excess thyroid hormone synthesis and secretion from the thyroid gland. Graves' disease (GD) is the most common cause of hyperthyroidism, characterized by unregulated stimulation of the TSH receptor by autoreactive TSH receptor antibodies (TRAb) (1). A rare etiology of hyperthyroidism is thyroid stimulating hormone (TSH) secreting pituitary adenoma (TSHoma), with the prevalence of 1–2 cases per million. Diagnosis of TSHoma should be considered when thyroid hormone levels increased without a suppressed TSH concentration, a condition referred to as SITSH (syndrome of inappropriate secretion of thyrotropin) (2). The concurrent of GD and TSHoma is rare. Here we report a case of GD and TSHoma with suppressed TSH levels, in which the coexistence of TSHoma could be easily misdiagnosed.

## Case Description

A 55-year-old Chinese female initially presented to our hospital in May 2019, complaining about 2-month history of thermophobia, anxiety, palpitation, hyperdefecation and weight loss. She denied other symptoms including headache, visual field defect or galactorrhea. She denied intakes of biotin and other medications. Physical examination showed that her body mass index was 22.8 kg/m^2^, blood pressure was 150/72 mmHg, and pulse was 110 bpm. The patient had mild upper eyelid retraction but no exophthalmos was observed. She had moist skin, tremors of hands and tongue, and a grade I goiter with soft texture. The patient had undergone hysterectomy for uterine leiomyomas 10 years ago. She denied family history of relevant disease including thyroid disease, pituitary disease, hypertension, coronary heart disease and diabetes mellitus.

Thyroid function test (Abbott, USA) showed FT_3_ 39 pmol/l (normal range, 2.63–5.7 pmol/l), FT_4_ 51.07 pmol/l (9.01–19.05 pmol/l) wisth TSH 0.337 mIU/l (0.35–4.94 mIU/l; intraassay coefficient of variation <6%). Her thyroid peroxidase antibodies (Abbott) and thyroglobulin antibodies (Abbott) were 1.56 IU/ml (0–5.61 IU/ml) and 197.7 IU/ml (0–4.11 IU/ml). Her TRAb (Roche, Switzerland), TSAb (Siemens Healthcare, Llanberis, UK) and thyroglobulin levels were 5.63 IU/l (0–1.75 IU/l), 4.7 IU/l (0–0.55 IU/l) and 56.6 ng/ml (1.6–59.9 ng/ml), respectively. Re-examination 1 week later showed FT_3_ 25.23 pmol/l, FT_4_ 39.25 pmol/l and TSH 0.2595 mIU/l. Similar results were obtained from different laboratories (Roche, Switzerland and Siemens, Germany). The mildly suppressed TSH levels were neither consistent with the condition of Graves' hyperthyroidism nor SITSH. Her blood test showed low level of neutrophils (Table 1). Thus, she was prescribed on propranolol and low iodine diet for symptomatic control and recommended for further assessments.

**Table 1:** Preoperative and postoperative results of baseline laboratory tests and hormone profiles.

**Parameter**	**Preoperative**	**Postoperative**	**Reference range**
**Baseline laboratory tests**			
WBC	4.10	7.00	3.50–9.50 × 10^9^/L
Neutrophil	0.78	3.54	1.80–6.30 × 10^9^/L
Lymphocyte	2.82	2.93	1.10–3.20 × 10^9^/L
HGB	132	124	115–150 g/L
RBC	3.99	4.45	3.80–5.10 × 10^12^/L
PLT	178	299	125–350 × 10^9^/L
K^+^	3.19	3.89	3.50–5.30 mmol/L
Na^+^	146.8	144.9	137.0–147.0 mmol/L
AST	28	21	13–35 (U/L)
ALT	33	31	7–40 (U/L)
GGT	106	141	7–45 (U/L)
TP	24	44	65–85 (g/L)
ALB	16.1	15.8	40–55 (g/L)
TBA	2	1	0–10 (umol/L)
TBIL	13	7.3	3.4–20.5 (umol/L)
Urea	5.82	4.25	2.85–7.14 (mmol/L)
Cr	32	34	45–84 (umol/L)
Cys-C	1.21	1.33	0.53–0.95 (mg/L)
**Hormone profiles**			
ACTH	37.76	33.59	7.2–63.3 (pg/ml)
Cortisol	489.9	406.9	Morning: 171–536 nmol/L Afternoon: 64–327 nmol/L
GH	0.32	0.88	0.05–8 ug/L
PRL	265	74.8	40–530 mIU/L
IGF-1	108	112	75–238 ng/ml
LH	21.3	17.5	11.3–39.8 mIU/mL
FSH	57	53	21.7–153 mIU/mL

*WBC, white blood cell; HGB, hemoglobin; RBC: red blood cell; PLT, platelet; Na^+^, sodium; K^+^, potassium; AST, aspartate transaminase; ALT, alanine transaminase; GGT, gamma-glutamyl transferase; TP, total protein; ALB, albumin; TBA, total bile acid; TBIL, total bilirubin; Cr, creatinine; Cys-C, Cystatin C; ACTH, adrenocorticotropin; GH, growth hormone; PRL, prolactin; IGF-1, Insulin-like growth factor-1; LH, Luteinizing hormone; FSH, Follicle stimulating hormone*.

Further assessments showed that sex hormone-binding globulin (SHBG) was elevated (158 nmol/l; 18–114 nmol/l). Her ACTH and growth hormone were normal, LH and FSH levels were concordant with her age (Table 1). After 24-h subcutaneous injection of octreotide (0.1 mg s.c.), TSH decreased from 0.11 to 0.04 mIU/l, the suppression ratio of TSH at 24 vs. 0 h was 63%. TRH stimulation was not performed due to unavailability of TRH in China. Mutations of the TSH receptor and thyroid hormone receptor β (*THRB*) genes were not identified.

**Figure 1:**
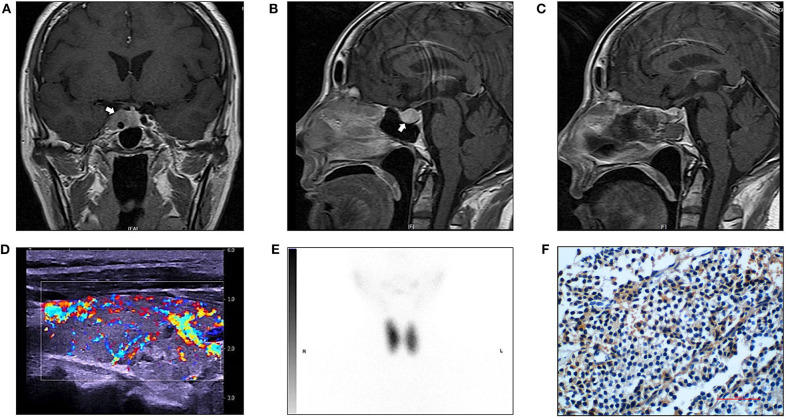
Radiology imaging of the patient and immunostaining for TSH in the pituitary tumor. **(A)** Preoperative magnetic (MR) resonance image (T1-weight, sagittal view). White arrow pointing to a less enhanced area in the sella (17 × 15 mm, arrow), involving the cavernous sinuses. **(B)** Preoperative magnetic (MR) resonance image (T1-weight, coronal view). **(C)** Postoperative MR image (T1-weight, sagittal view). **(D)** Thyroid ultrasound revealed a heterogenous parenchyma with rich blood flow and a nodule (ACR TR 3) located in the left lobe. The right thyroid lobe measures 5.00 × 1.70 × 1.87 cm. The thyroid isthmus measures 0.23 cm in AP dimension. The left thyroid lobe measures 5.08 × 1.77 × 1.97 cm. **(E)** Radionuclide (^99^mTcO_4_) scan showed diffusely increased uptake, the ratio of uptake of the tracer in the thyroid to that in the background was 45 (right lobe) and 40 (left lobe). **(F)** Positive immunostainning for TSH in the pituitary tumor (magnification 400 ×).

Magnetic resonance imaging (MRI) displayed a less enhanced area in the sella (17 × 15 mm), involving the cavernous sinuses (Figures 1A,B). Increased blood flow in the thyroid was detected in ultrasound, with a nodule (ACR TR 3) located in the left lobe (Figure 1D). Radionuclide scan (^99^mTcO_4_) showed diffusely increased uptake (Figure 1E). Computer tomography scan (CT) showed normal adrenal glands.

Based on clinical and biochemical evidence for hyperthyroidism, low but incompatibly suppressed TSH, elevated TRAb, TSH suppression by octreotide, and the MRI finding, a diagnosis of TSHoma with concomitant GD was highly suspected.

In July 2019, the pituitary mass was removed through the endoscopic trans-sphenoidal resection. To prevent thyroid storm, the patient was treated with propranolol pre-operation, and we made thorough preparation with a team of experienced neurosurgeons, endocrinologists and unit of anesthesia and intensive care. The patient experienced an uneventful surgery and postoperative recovery. Surgical pathology assessment confirmed a pituitary macroadenoma. Immunohistochemistry showed positive staining for TSH (mouse monoclonal to TSH, Thermo Fisher Scientific, Fremont, CA, USA) (Figure 1F), whereas negative staining for ACTH, GH or PRL. The percentage of positive Ki-67 was 1%. Serum TSH was 0.001 mIU/l at 1 week after the surgery. These findings confirmed the presence of TSHoma.

**Figure 2:**
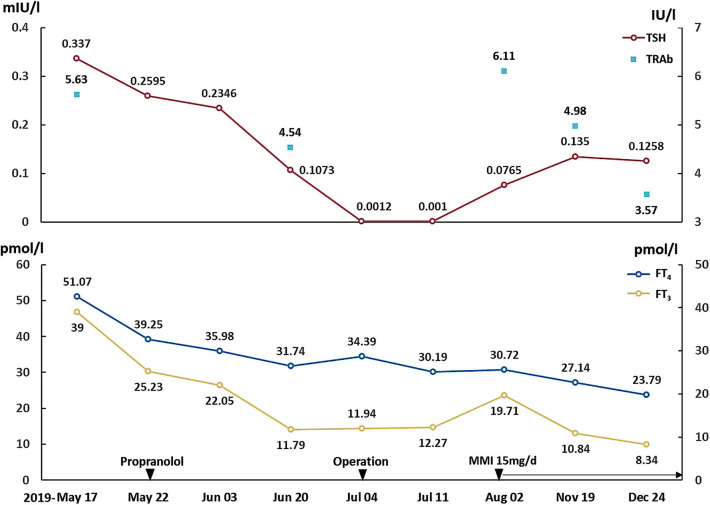
Changes of TSH, FT_3_, FT_4_, and TRAb. Propranolol was prescribed on May 22, 2019. The operation was performed on July 4, 2019, and the methimazole was started on August 2, 2019. TSH, thyroid-stimulating hormone; FT_3_, free triiodothyronine; FT_4_, free thyroxine; TRAb, TSH receptor antibodies.

After surgery, her symptoms got alleviated but did not vanish. The patient did not have exophthalmos after surgery. Post-operation follow-up at 1 month showed that her FT_3_ was 19.71 pmol/l, FT_4_ was 30.72 pmol/l, TSH was 0.0765 mIU/l, TRAb was 6.11 IU/l and TSAb was 5.58 IU/l (Figure 2). This was supportive for an untreated GD. She was prescribed on methimazole 15 mg/day and propranolol 47.5 mg/day. In December 2019, she reported 4 kg-weight gain and improved symptoms. Her FT_3_ and FT_4_ decreased to 8.34 and 23.79 pmol/l, respectively, and TSH increased to 0.1258 mIU/l (Figure 2). TRAb and TSAb was declined to 3.57 and 1.45 IU/l. The MRI did not show any sign of tumor regrowth (Figure 1C). She remained on methimazole 15 mg/day and was followed up.

## Discussion

TSHoma is a rare disease accounting for 0.5–3% of all functioning pituitary tumors (3). The first case of TSHoma was reported in 1960 (4). Clinical features of TSHoma are often characterized by mild signs and symptoms of thyrotoxicosis and neurological symptoms caused by the compression of pituitary mass (2). The diagnosis of TSHoma is usually based on the biochemical findings of SITSH, histochemical and imaging evidence. SHBG and THRβ mutation should be examined to exclude the possibility of RTH.

TSHoma complicated by GD is extremely rare. There have been only 13 cases reported so far (Table 2) (5–16). Among them, six cases were found the coexistence of TSHoma and GD upon the initial diagnosis (6, 9, 13–16). Majority of these cases presented normal to elevated TSH with increased thyroid hormones, which was suggestive of SITSH, therefore the possibility of TSHoma was checked. In the present case, repeated TSH measurements were below the normal range. Given her predominant manifestations of thyrotoxicosis and positive TRAb, the condition could be easily misdiagnosed as GD alone. Nonetheless, keeping in mind that the TSH level in untreated overt GD is normally suppressed to below 0.1 mU/l by negative feedback due to obviously elevated thyroid hormones, we identified the hint for her unusual etiology of hyperthyroidism. We suspected the possibility of coexistent central hyperthyroidism instead of initiating antithyroid medication. Further investigation and surgical pathology confirmed our diagnosis.

**Table 2:** Case reports of patients with GD and TSHoma.

**References**	**Country**	**Age/gender**	**Clinical presentation**	**TSH(mIU/L)**	**Pituitary tumor size (mm)**	**Clinical course**	**Treatment**	**Follow up of TSHoma**
O'Donnell et al. (5)	UK	25/M	Signs of thyrotoxicosis, peripheral vision defect	28	N/A	TSHoma → GD	Cortisol, testosterone, antithyroid drug, thyroxine, hypophysectomy	TSH remained undetectable
Sandler (6)	USA	56/F	Signs of thyrotoxicosis, acromegaly, symptomatic ophthalmopathy	8.1	N/A	GD complicated with TSHoma	Antithyroid drug, cortisone, pituitary irradiation, radioiodine therapy	Recurrence of hyperthyroidism
Azukizawa (7)	N/A	N/A	N/A	N/A	N/A	TSHoma → GD	N/A	N/A
Kamoi et al. (8)	Japan	46/F	Signs of thyrotoxicosis and galactorrhoea	15.5	N/A	TSHoma → GD	Antithyroid drug, prednisolone, transsphenoidal surgery	Recurrence of hyperthyroidism
Koriyama et al. (9)	Japan	31/F	Signs of thyrotoxicosis	2.1	N/A	GD complicated with TSHoma	Octreotide and levothyroxine sodium, antithyroid drug, transsphenoidal surgery (twice)	Discontinue treatment for financial reasons
Kageyama et al. (10)	Japan	21/F	Signs of thyrotoxicosis	3.16	10	TSHoma → GD	Transsphenoidal surgery	N/A
Lee et al. (11)	China	27/M	Signs of thyrotoxicosis	0.004	10.4	GD → TSHoma	Antithyroid drug, transsphenoidal surgery	N/A
Lee et al. (11)	China	28/F	Signs of thyrotoxicosis	0.123	15	GD → TSHoma	Antithyroid drug	N/A
Ogawa et al. (12)	Japan	32/F	Signs of thyrotoxicosis	Less than detectable	5	GD → TSHoma	Antithyroid drug, transsphenoidal surgery	Recovery
Kamoun et al. (13)	France	36/F	Signs of thyrotoxicosis, exophthalmos	1.2–1.8	10	GD complicated with TSHoma	Antithyroid drug, lanreotide, thyroid lobectomy, transsphenoidal surgery	Recovery
Okuyucu et al. (14)	Turkey	37/F	Signs of thyrotoxicosis, goiter and exophthalmos	5.54	13	GD complicated with TSHoma	Thyroidectomy, transsphenoidal surgery	N/A
Arai et al. (15)	Japan	40/F	Signs of thyrotoxicosis	0.27	13	GD complicated with TSHoma	Antithyroid drug, transsphenoidal surgery	Recovery
Li et al. (16)	China	55/M	Signs of thyrotoxicosis, recurrent atrial fibrillation	8.9	23	GD complicated with TSHoma	Antithyroid drug, transsphenoidal surgery	Recurrence of TSHoma
Present case	China	55/F	Signs of thyrotoxicosis	0.337	17	GD complicated with TSHoma	Transsphenoidal surgery, antithyroid drug	Recovery

*F, Female; M, male; GD, Graves' disease; N/A, Not available*.

Whether it is an incidental coexistence of TSHoma with GD or there is a mechanism that connects TSHoma and GD remained to be elucidated. Both the continuous elevation or fluctuation of TSH might induce the production of anti-idiotypic antibodies, or regulate Fas-mediated apotosis against the thyroid gland, leading to GD (8, 9). More evidence is needed to confirm the association between GD and TSHoma.

TSHoma is characterized by expression of somatostatin receptors (SSTRs), especially SSTR2 and SSTR5. Administration of long acting somatostatin analogs such as octreotide for at least 2 months is useful in the differential diagnosis of central hyperthyroidism, as well as the treatment of TSHoma (17). One case also reported successful treatment of TSHoma with pasireotide, as an alternative somatostatin analog (18). A recent study suggested that somatostatin analog (SSA) suppression test was able to diagnose TSHoma with a positive predictive value of 88.89%. The test also showed good performance in distinguishing RTH and TSHoma without imaging evidence, which is valuable for medical centers unable to conduct mutation analysis (19). This suppression would be easily recognized if the basal TSH is normal or high. The present report, for the first time, presented the effect of octreotide on the diagnosis of TSHoma and GD with slightly suppressed TSH level.

As we all know, the treatment of TSHoma is very different from that of GD. Thus, it is challenging to treat TSHoma concomitant GD. We noticed in previous cases, using anti-thyroid drugs as the first-choice treatment led to the risk of deterioration of thyroid function (8, 11, 12, 15). It is not surprising as antithyroid drugs might increase the level of TSH by inhibiting the negative feedback, thus stimulating the growth of TSHoma. Besides, the patient showed low neutrophil levels before surgery, which is the contradiction of antithyroid drugs. Hence, we prescribed beta-receptor blocker instead of methimazole before her neurosurgery. Indeed, based on the patient's positive result of octreotide suppression test, we had considered to use long-acting form of somatostatin analogs pre-operatively as the medication might relive her symptoms of hyperthyroidism and shrinking the size of pituitary tumor (3), but the patient refused due to its high cost.

Interestingly, the patient showed temporary remission after taking propranolol. There were two possible explanations suspected. First, the level of TSH secreted by TSHoma is fluctuating, which might occur even without medication. The decreased TSH led to decreased FT_3_ and FT_4_. Second, propranolol inhibits the conversion from T_4_ to T_3_ (20), therefore it might exert some beneficial effects in thyrotoxicosis (21). The combination of low iodine diet and propranolol was effective in controlling the hyperthyroidism of our patient in a short time, which could be inspiration in the management of patients like this case.

In this challenging case, we failed to normalize the thyroid function of the patient pre-operatively. Even though thyroid storm is rarely reported in pituitary surgery (2), we did thorough plan with a multidisciplinary team to prevent this event. After surgery, we initiated methimazole treatment for her persistent GD and kept watching her clinical manifestation, thyroid function and pituitary imaging.

Limitations in this report include: First, we did not detect the level of α-TSH. Elevated FSH level was common for postmenopausal women, which may interfere with the detection of TSH (22). Unfortunately, we were unable to perform this test due to the unavailability of the test kit of alpha-subunits. Second, we did not perform the TRH stimulation test limited by detection methods, therefore the information relating to HPA axis function was inadequate. Alternatively, we tested pituitary hormones both before and after surgery and the results were normal. Third, the expression of SSTR2/SSTR5, which was useful in deciding whether SSA can be potential treatment, was not examined in this case. This examination is not a routine test in our hospital, so we did the octreotide suppression test instead, which indicated that SSA might be useful in reducing the growth and treating the recurrence of TSHoma.

In conclusion, this report described a patient who had clear biochemical and clinical features of hyperthyroidism with mildly suppressed TSH levels. Further investigations revealed the dual presence of TSHoma and GD. This case reminds clinicians that careful interpretation of thyroid function test is important during the diagnosis of hyperthyroidism. It also highlights the challenges in the diagnosis and treatment of this rare condition.

## Ethics Statement

Written informed consent was obtained from the individual for the publication of any potentially identifiable images or data included in this article.

## Author Contributions

HG and XW designed the study, collected data, and managed the patients. JF prepared the manuscript and figures. AW operated on and followed up the patient. HG revised the manuscript. All authors contributed to the article and approved the submitted version.

## Conflict of Interest

The authors declare that the research was conducted in the absence of any commercial or financial relationships that could be construed as a potential conflict of interest.
